# Prokaryotic ribosomal RNA stimulates zebrafish embryonic innate immune system

**DOI:** 10.1186/s13104-019-4878-8

**Published:** 2020-01-03

**Authors:** Abhishikta Basu, Maki Yoshihama, Tamayo Uechi, Naoya Kenmochi

**Affiliations:** Frontier Science Research Center, University of Miyazaki, 5200 Kihara, Kiyotake, Miyazaki, 889-1692 Japan

**Keywords:** rRNA, Lipopolysaccharide, MAMP, Zebrafish, Immunogenicity

## Abstract

**Objectives:**

Cell-culture studies reported that prokaryotic RNA molecules among the various microbe-associated molecular patterns (MAMPs) were uniquely present in live bacteria and were categorized as viability-associated MAMPs. They also reported that specific nucleotide modifications are instrumental in the discrimination between self and nonself RNAs. The aim of this study was to characterize the in vivo immune induction potential of prokaryotic and eukaryotic ribosomal RNAs (rRNAs) using zebrafish embryos as novel whole animal model system. Additionally, we aimed to test the possible role of rRNA modifications in immune recognition.

**Results:**

We used three immune markers to evaluate the induction potential of prokaryotic rRNA derived from *Escherichia coli* and eukaryotic rRNAs from chicken (nonself) and zebrafish (self). Lipopolysaccharide (LPS) of *Pseudomonas aeruginosa* served as a positive control. *E. coli* rRNA had an induction potential equivalent to that of LPS. The zebrafish innate immune system could discriminate between self and nonself rRNAs. Between the nonself rRNAs, *E. coli* rRNA was more immunogenic than chicken rRNA. The in vitro transcript of zebrafish 18S rRNA gene without the nucleotide modifications was not recognized by its own immune system. Our data suggested that prokaryotic rRNA is immunostimulatory in vivo and could be useful as an adjuvant.

## Introduction

The recognition of microbe-associated molecular patterns (MAMPs) evokes the host innate immune system to induce downstream signaling pathways to eliminate the microbe. Among MAMPs, prokaryotic RNA molecules are distinctly called viability-associated MAMPs (*vita*-MAMPs) because of their unique association with live microbes. Since the innate immune system can discriminate live microorganisms from dead ones via recognition of *vita*-MAMPs, studies on immunostimulation potential of *vita*-MAMPs have attracted attention in the fields of vaccines and adjuvants [1]. The immune response induced by bacterial RNA upon recognition includes NF-*κ*B-dependent proinflammatory cytokines, type I interferons and inflammasome activation [2]. In vitro studies suggested that RNA modifications could silence immune response and could contribute in the discrimination between self and nonself RNAs [3–5]. Eukaryotic ribosomal RNA (rRNA) possesses extensive modifications than prokaryotic rRNA. For example, human rRNA has at least 10 times more pseudouridine (Ψ) and 25 times more 2′-*O*-methylated nucleotides than bacterial rRNA [3]. However, the role of rRNA in immune recognition is still underexplored.

In this study, we introduced zebrafish embryos as whole animal model system to characterize the in vivo induction potential of self and nonself (prokaryotic and eukaryotic) rRNAs. In addition, we compared the induction potentials of the in vitro transcribed zebrafish 18S (IVT-18S) rRNA (devoid of any modifications) and native self rRNA to test the possible role of rRNA modifications in immune recognition. Zebrafish embryo is an attractive model system due to its small size, rapid life cycle, ex utero development, optical transparency and many other advantages [6]. We compared the induction potentials of the different rRNAs in zebrafish embryos with that of lipopolysaccharide (LPS) of *Pseudomonas aeruginosa*. LPS is a potent classic MAMP that is associated with the bacterial cell wall of both live and dead microbes. Our data demonstrated that zebrafish embryonic innate immune system could discriminate between externally administered self and nonself rRNAs, allowing induction of immune response specifically against the nonself rRNAs. The nonself prokaryotic rRNA was more potent stimulant than the nonself eukaryotic rRNA. Furthermore, the IVT-18S rRNA was not recognized by its own immune system. Taken together, our work reproduced some of the in vitro results of *vita*-MAMPs in a novel in vivo model using rRNA as the stimulant and suggested the possible use for prokaryotic rRNA in vaccines as adjuvant.

## Main text

### Methods

#### Purification of rRNA

The rRNAs were derived from *Escherichia coli* (nonself prokaryotic), chicken (nonself eukaryotic) and zebrafish (self). Liver from freshly sacrificed chicken (*Gallus gallus domesticus*) was purchased from a local meat shop. Zebrafish (*Danio rerio*) embryos were obtained by breeding the adults of AB line at the animal house facility of University of Miyazaki following the animal welfare regulations (Authorization number: 2018-531). Total RNA was extracted from *E. coli* HST08 competent cells (TAKARA) (cultured for 2.5 h) using the hot-phenol method and that from chicken liver and zebrafish embryos using TRI-reagent (Molecular Research Center Inc., USA). NucleoBond RNA/DNA 400 kit (Macherey-Nagel, Germany) was used to purify rRNA from total RNA. Following purification, rRNA was pelleted by isopropanol precipitation. The pellet was resuspended in water and quantified by NanoDrop measurement. Purity was confirmed by ratios of 260/280 and 260/230 > 1.9. The quality of the rRNA was assessed in MOPS denaturing gel (Additional file 1: Fig. S1).

#### In vitro transcription

Genomic DNA was extracted by heating one zebrafish embryo in 50 mM NaOH at 95 °C for 10 min. LA Takara kit and T7 promoter containing specific primers were used to amplify 18S rRNA region (Additional file 3: Table S1). Approximately 500 ng of the PCR product was in vitro transcribed using the T7-Scribe Standard RNA IVT kit (Cellscript, USA).

#### Zebrafish embryos and microinjection

Adult breeders of AB lines were reared following the standard guidelines [7]. Zebrafish embryos at 30 h post fertilization (hpf) were anaesthetized in E3 medium containing 0.3 mg/ml tricaine methanesulfonate (Research Organics, USA) and then mounted onto presolidified 1% agarose gel-bed for microinjection. Test stimulants of ~ 5.7 ng/nl concentration were injected into the yolk sac of embryos using IM-30 micromanipulator (Narishige, Japan). For the dose of 40 ng, ~ 7 nl volume was injected. Injection volume was measured by the standard micrometry method. Our experimental design is schematically represented in Additional file 2: Fig. S2. We used the induction levels of water-injected embryos as a control to quantitate the induction levels of test stimulants at the respective time points. Control embryos were injected with ~ 7 nl of water. Injected embryos were maintained in sterile E3 medium at 28.5 °C until the incubation period. A batch of injected embryos was kept for observation until 3 days post injection (3 dpi).

#### RNA quantitation

Total RNA was extracted from a pool of eight embryos from three biological replicates. Approximately 1 μg DNase-treated total RNA was reverse transcribed into cDNA using High-capacity cDNA Reverse Transcription kit (Applied Biosystems, USA). PCR was performed with a TAKARA thermocycler using Prime Taq DNA polymerase (GeNet Bio, Korea) and gene-specific primers for 35 cycles. Additional file 3: Table S1 provides the primer details of interleukin 8 gene (*il8*), pro-interleukin 1β gene (*il1β*), proteasome subunit β9a gene (*psmb9a*) and ribosomal protein L38 gene (*rpl38*) used in this study. PCR products were run in 1.5% agarose gel electrophoresis using TAE buffer. Real-time or quantitative PCR (qPCR) was performed using Power SYBR Green PCR Master Mix (Applied Biosystems, USA) and the same primers in StepOnePlus Real-time PCR system (Applied Biosystems, USA). Changes in the transcript levels were normalized to that of *rpl38* and expressed as fold changes relative to control using the ∆∆Ct method. Student’s t-test was applied to calculate *p*-value, and a value of < 0.05 was considered statistically significant.

### Results

#### Bacterial rRNA shows immunogenicity similar to LPS

Embryos were injected at 30 hpf with various stimulants, and their induction potentials were compared at 2, 6 and 24 hpi by measuring the induced expression levels of *il8*, *il1β* and *psmb9a* genes. The markers IL8 and IL1β represent proinflammatory cytokines. Briefly, IL8 is a potent chemokine, and IL1β drives the expression of genes required for immune-mediated inflammation, effective adaptive immunity and antiviral control [8]. The other marker PSMB9A (also known as low molecular mass polypeptide 2 or LMP2) is a downstream target of interferon γ [9] and is involved in MHC class I antigen presentation.

**Fig. 1:**
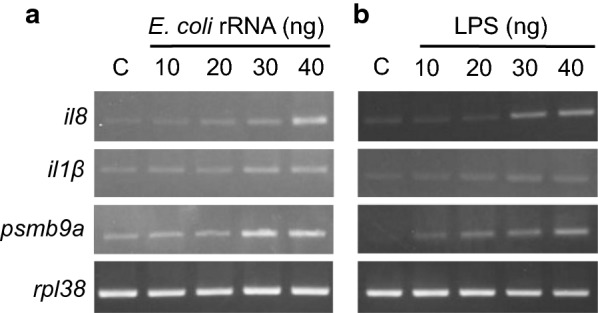
Dose-dependent increase in the expression levels of markers upon *E. coli* rRNA and LPS stimulations. *E. coli* rRNA (10–40 ng) **a** and LPS (10–40 ng) **b** were injected into zebrafish embryos at 30 hpf. At 6 hpi, the induced expression levels of *il8*, *il1β*, *psmb9a* and *rpl38* were analyzed by PCR for the respective doses of stimulants. *C* Control (water)

To determine a suitable stimulation dose, expression levels of immune markers at different amounts of *E. coli* rRNA and LPS were tested. At 40-ng dose, both *E. coli* rRNA and LPS stimulations induced enhanced expression of immune markers compared with control (Fig. 1). Additionally, the injected embryos of both the stimulants at this dose survived the observation period of 3 dpi without any abnormalities (data not shown). Therefore, 40 ng was standardized as the stimulation dose in this study.

**Fig. 2:**
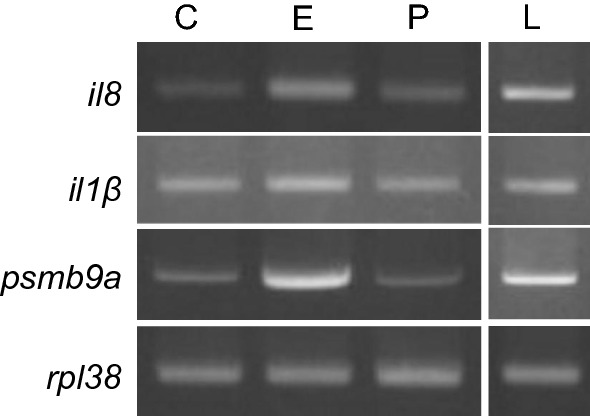
Comparative induction potential of *E. coli* rRNA, poly (I:C) and LPS. Upon microinjection with 40 ng of stimulants, the induced expression levels of *il8*, *il1β*, *psmb9a* and *rpl38* were analyzed at 6 hpi by PCR. *C* Control (water), *E E. coli* rRNA, *P* Poly (I:C) and *L* LPS

When the induction potential of *E. coli* rRNA was compared with that of poly (I:C) and LPS, *E. coli* rRNA upregulated transcription of *il8* and *psmb9a* similar to that of LPS (Fig. 2). Induced levels of *il1β* were inconclusive. In contrast, the expression levels of all the markers induced by poly (I:C) were similar to that of control. This result demonstrated that the immune response induced upon *E. coli* rRNA stimulation at the given conditions was similar to that of LPS and robust compared with that of poly (I:C). Therefore, we excluded poly (I:C) from further experiments and used only LPS as a positive control.

**Fig. 3:**
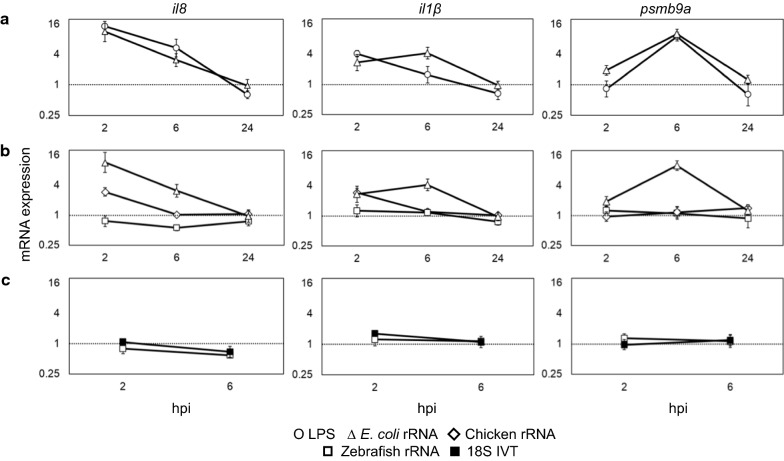
Comparative induction potentials of LPS; rRNAs from *E. coli*, chicken and zebrafish; and IVT-18S rRNA. Upon microinjection with 40 ng of various stimulants, the induced levels of *il8*, *il1β* and *psmb9a* expression were estimated by qPCR at indicated times. Induction potential was compared between *E. coli* rRNA and LPS **a** among *E. coli*, chicken and zebrafish rRNAs **b** and between IVT-18S and native self rRNAs **c**. Induction levels of water-injected embryos (represented as fold 1 in the figures) were used as a control to quantitate the induction levels of test stimulants at the respective time points. Values are mean ± SE (n = 3)

To validate our results, we quantitated the levels and studied the kinetics of induction upon *E. coli* rRNA and LPS stimulations in time-course experiments (Fig. 3a). *E. coli* rRNA and LPS displayed similar levels and kinetics of *il8* and *psmb9a* induction, peaking at 2 and 6 hpi, respectively. In contrast, *il1β* induction was highest at 2 hpi for LPS and 6 hpi for *E. coli* rRNA with same induction values. However, the induction levels at the respective time points were not significantly different between the two stimulants. Induction of all the markers by both the stimulants resolved by 24 hpi. Taken together, *E. coli* rRNA had equivalent immunogenicity as that of LPS in zebrafish embryos.

#### Zebrafish embryos can discriminate between self and nonself rRNAs

Next, we compared the induction levels and kinetics of zebrafish (self), chicken (nonself eukaryotic) and *E. coli* (nonself prokaryotic) rRNAs (Fig. 3b). Self rRNA did not induce any of the markers within the experimental time frame, implying that it skipped the immune recognition as expected. Chicken rRNA induced *il8* and *il1β* expressions only at 2 hpi followed by resolution approximately 6 hpi. It did not induce *psmb9a* expression. Comparing the immunogenicity of the two nonself rRNAs, *E. coli* rRNA induced three markers for 24 h, whereas chicken rRNA induced only two for approximately 6 h. At 2 hpi, the induced expression level of *il8* was higher for *E. coli* rRNA than chicken rRNA, but the values were not significantly different. On the other hand, the expression levels of *il1β* induction at 2 hpi were same for both the nonself rRNAs (Fig. 3b). Taken together, the zebrafish embryonic innate immune system could discriminate between self and nonself rRNAs, and the immunogenicity of nonself prokaryotic rRNA was robust compared with nonself eukaryotic rRNA.

#### In vitro transcribed self 18S rRNA did not stimulate zebrafish embryos

Previous studies with oligoribonucleotides and transfer RNA (tRNA) reported that the presence of 2′-*O*-methylated nucleotides and pseudouridine (ψ) could silence immune response [3, 4]. To test whether nucleotide modifications of rRNA were involved in the discrimination of self rRNA, we generated the IVT-18S rRNA devoid of any modifications. Since 2 and 6 hpi were more critical in previous kinetics studies, we compared the induction potentials of IVT-18S rRNA and native self rRNA at these time points (Fig. 3c). Unexpectedly, IVT-18S rRNA did not show any induction, as was observed in the case of native self rRNA. This result suggested that under the given conditions, the zebrafish innate immune system could not recognize either modified or unmodified self 18S rRNA as an immunostimulant.

### Discussion

In this study, we found that induction of *il8* upon stimulation was rapid, which correlated with the rapid *il8* induction during acute inflammation reported in zebrafish larvae [10]. The peak induction of *psmb9a* at 6 hpi upon LPS stimulation was demonstrated previously [11]. Since the molecules involved in antigen processing are transcriptionally induced, even though adaptive immunity was not developed in zebrafish embryos [12], the same level of *psmb9a* induction at 6 hpi by both *E. coli* rRNA and LPS indicated their corresponding ability to induce adaptive immunity. The kinetics of *il1β* induction by live *E. coli* infection [13] precisely correlated with our data, thereby underscoring the immunogenicity of rRNA in zebrafish embryos.

The LPS-induced kinetics of *il1β* expression in our results were different from that of heat-killed *E. coli* stimulation [13], suggesting that the stimulation potential of individual MAMPs could be different from that of a combination of MAMPs presumably present in the heat-killed *E. coli* suspension. IL1β secretion is associated with bacterial viability [1]. Whether the prolong transcription of *il1β* induced by prokaryotic rRNA (*vita*-MAMP) compared with LPS (classic MAMP) as indicated by their different temporal peaks is a signature for viability sensing needs to be determined.

The robust immunogenicity of *E. coli* rRNA compared with chicken rRNA was corroborated by their respective antiviral activity [14]. Similar contrast in the induction potential of prokaryotic and eukaryotic RNA was also demonstrated between *E. coli* and mammalian total RNA [3] and tRNA [4]. Together, they confirmed that nonself prokaryotic rRNA was more potent than nonself eukaryotic rRNA. While the result of no induction upon stimulation by self rRNA was expected, that for IVT-18S rRNA was not. Although further investigation is required to confirm the role of nucleotide modifications, our findings suggested that under the given experimental conditions, nucleotide modifications of 18S rRNA were not involved in the discrimination of self rRNA. Immune discrimination between self and nonself nucleic acids is not restricted to nucleotide modifications [4]. The immune system might discriminate self 18S rRNA from the nonself by any particular conformation or sequence. Since the modifications within a specific sequence of *E. coli* 23S rRNA was reported to abolish immune induction [2], the possible involvement of modifications in zebrafish 28S rRNA needs to be investigated.

Monophosphoryl lipid A is a nontoxic derivative of LPS used as a licensed adjuvant for human use [1]. The overall similarities between the immunogenicity of *E. coli* rRNA and LPS in the present study together with the reported antiviral activity of *E. coli* rRNA [14] indicated that prokaryotic rRNA could have LPS-like adjuvant potential. The use of nonvirulent prokaryotic rRNA could be advantageous in augmenting the immunogenicity of live attenuated vaccines without the associated safety risks. Therefore, further examination determining the efficacy of prokaryotic rRNA as an adjuvant is worthy of consideration.

## Limitations

In this study, we tested the induction potential of prokaryotic rRNA derived from only one source. Inclusion of other bacterial sources like Gram positive bacteria will widen the survey. We used three genes as the representative markers to evaluate the immune potential. Combination with the whole transcriptome analysis will improve our understanding of global immune induction. We tested induction only at transcription level. Testing the induction at translation level will substantiate our data. We used the unmodified in vitro transcribed 18S zebrafish rRNA to investigate the role of nucleotide modifications in immunostimulation. This synthetic RNA partially represents the ideal unmodified version of native rRNA.

## Supplementary information

**Additional file 1: Fig. S1.** MOPS gel shows the qualities of stimulant ribosomal rRNAs. Before microinjection, 100 ng of column purified rRNAs from *E. coli,* zebrafish and chicken were run in 1.5% MOPS denaturing agarose gel to assess their qualities.

**Additional file 2: Fig. S2.** The experimental design is represented schematically. Zebrafish embryos of AB line were treated with 0.003% bleach (Kao, Japan) to remove germs and raised in sterile E3 medium at 28.5 °C. At 24 hpf, the chorion layer was torn off (dechorionated). Embryos were injected at 30 hpf with various samples, including LPS of *Pseudomonas aeruginosa* (Sigma, USA), poly (I:C) (Sigma, USA), water (control) and rRNAs of *E. coli*, chicken and zebrafish. Total RNA was extracted from a pool of 8 injected embryos at indicated time points to assess the induction level upon stimulation by qPCR. RT: Reverse transcription.

**Additional file 3: Table S1.** Primer sequences of immune markers and in vitro transcribed RNA.

## Data Availability

All data generated or analysed during this study are included in this published article and its supplementary information files. There isn’t any sequencing data for sharing at the moment as no datasets have been generated or analyzed.

## Abbreviations

MAMPs: microbe-associated molecular patterns
*vita*-MAMPs: viability-associated MAMPs
hpf: hours post fertilization
hpi: hours post injection
rRNA: ribosomal RNA
tRNA: transfer RNA
LPS: lipopolysaccharide
