# Supplementary material for: Prokaryotic ribosomal RNA stimulates zebrafish embryonic innate immune system
Source: BMC Res Notes. 2020 Jan 3;13:6. doi: 10.1186/s13104-019-4878-8 (PMC6942260; doi:10.1186/s13104-019-4878-8)
Supplement: Supplementary file 1 — Additional file 1: Fig. S1. MOPS gel shows the qualities of stimulant ribosomal rRNAs. Before microinjection, 100 ng of column purified rRNAs from E. coli, zebrafish and chicken were run in 1.5% MOPS denaturing agarose gel to assess their qualities. [file 13104_2019_4878_MOESM1_ESM.pptx]

## Slide 1
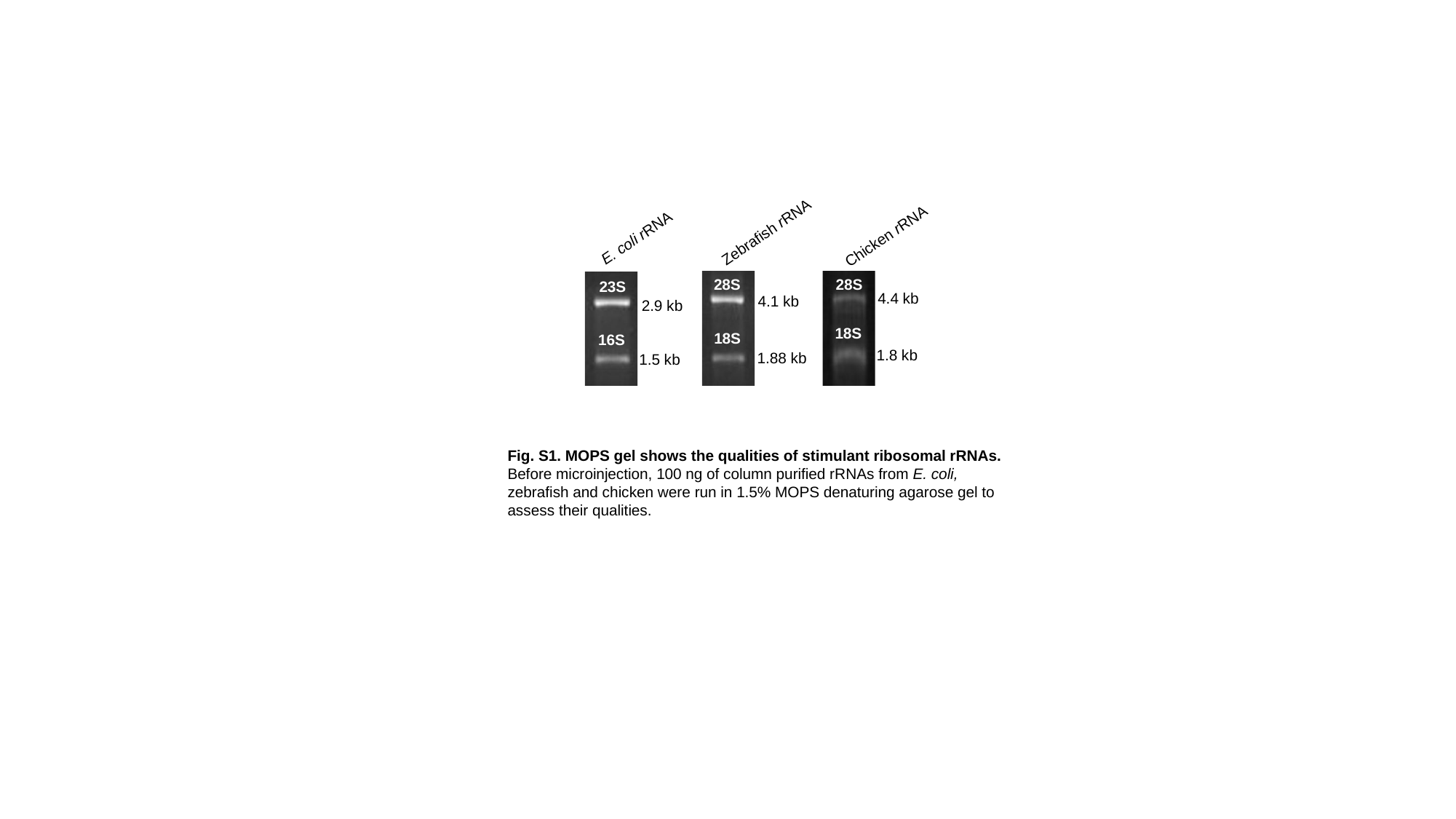

Zebrafish rRNA
Chicken rRNA
E. coli rRNA
28S
28S
23S
4.4 kb
4.1 kb
2.9 kb
18S
18S
16S
1.8 kb
1.88 kb
1.5 kb
Fig. S1. MOPS gel shows the qualities of stimulant ribosomal rRNAs.
Before microinjection, 100 ng of column purified rRNAs from E. coli, zebrafish and chicken were run in 1.5% MOPS denaturing agarose gel to assess their qualities.
