# Supplementary material for: Prokaryotic ribosomal RNA stimulates zebrafish embryonic innate immune system
Source: BMC Res Notes. 2020 Jan 3;13:6. doi: 10.1186/s13104-019-4878-8 (PMC6942260; doi:10.1186/s13104-019-4878-8)
Supplement: Supplementary file 2 — Additional file 2: Fig. S2. The experimental design is represented schematically. Zebrafish embryos of AB line were treated with 0.003% bleach (Kao, Japan) to remove germs and raised in sterile E3 medium at 28.5 °C. At 24 hpf, the chorion layer was torn off (dechorionated). Embryos were injected at 30 hpf with various samples, including LPS of Pseudomonas aeruginosa (Sigma, USA), poly (I:C) (Sigma, USA), water (control) and rRNAs of E. coli, chicken and zebrafish. Total RNA was extracted from a pool of 8 injected embryos at indicated time points to assess the induction level upon stimulation by qPCR. RT: Reverse transcription. [file 13104_2019_4878_MOESM2_ESM.pptx]

## Slide 1
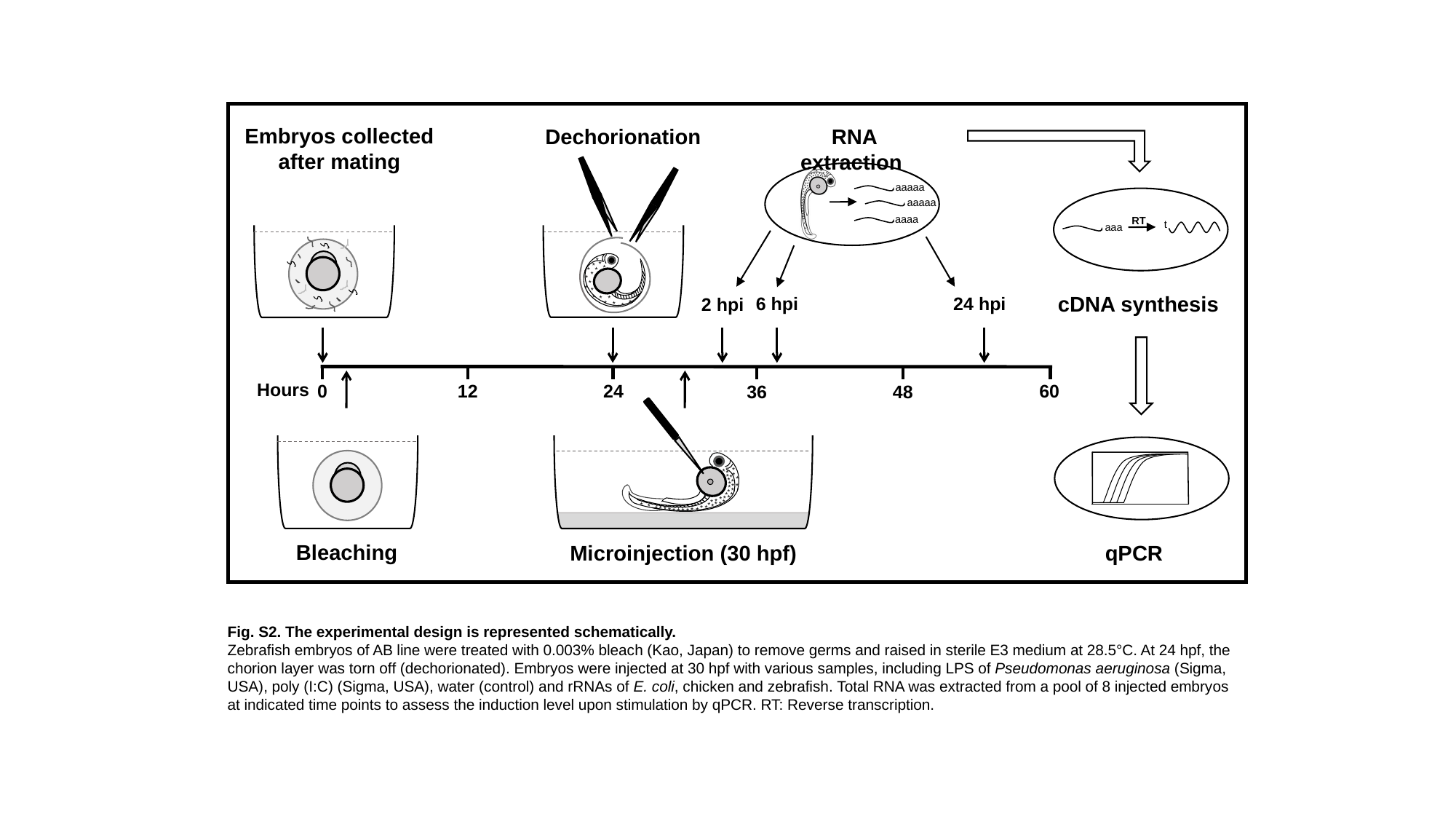

Embryos collected after mating
Dechorionation
RNA extraction
aaaaa
aaaaa
aaaa
RT
t
aaa
*
*
*
*
*
*
*
*
*
*
*
cDNA synthesis
6 hpi
24 hpi
*
2 hpi
*
*
*
*
*
*
Hours
12
60
0
24
36
48
*
*
*
*
*
*
*
*
*
*
*
*
*
*
*
*
*
*
*
*
*
*
*
*
*
*
*
*
*
*
*
*
*
*
*
*
*
*
*
*
*
Bleaching
qPCR
Microinjection (30 hpf)
Fig. S2. The experimental design is represented schematically.
Zebrafish embryos of AB line were treated with 0.003% bleach (Kao, Japan) to remove germs and raised in sterile E3 medium at 28.5°C. At 24 hpf, the chorion layer was torn off (dechorionated). Embryos were injected at 30 hpf with various samples, including LPS of Pseudomonas aeruginosa (Sigma, USA), poly (I:C) (Sigma, USA), water (control) and rRNAs of E. coli, chicken and zebrafish. Total RNA was extracted from a pool of 8 injected embryos at indicated time points to assess the induction level upon stimulation by qPCR. RT: Reverse transcription.
