# Supplementary material for: Prokaryotic ribosomal RNA stimulates zebrafish embryonic innate immune system
Source: BMC Res Notes. 2020 Jan 3;13:6. doi: 10.1186/s13104-019-4878-8 (PMC6942260; doi:10.1186/s13104-019-4878-8)
Supplement: Supplementary file 3 — Additional file 3: Table S1. Primer sequences of immune markers and in vitro transcribed RNA. [file 13104_2019_4878_MOESM3_ESM.pptx]

## Slide 1
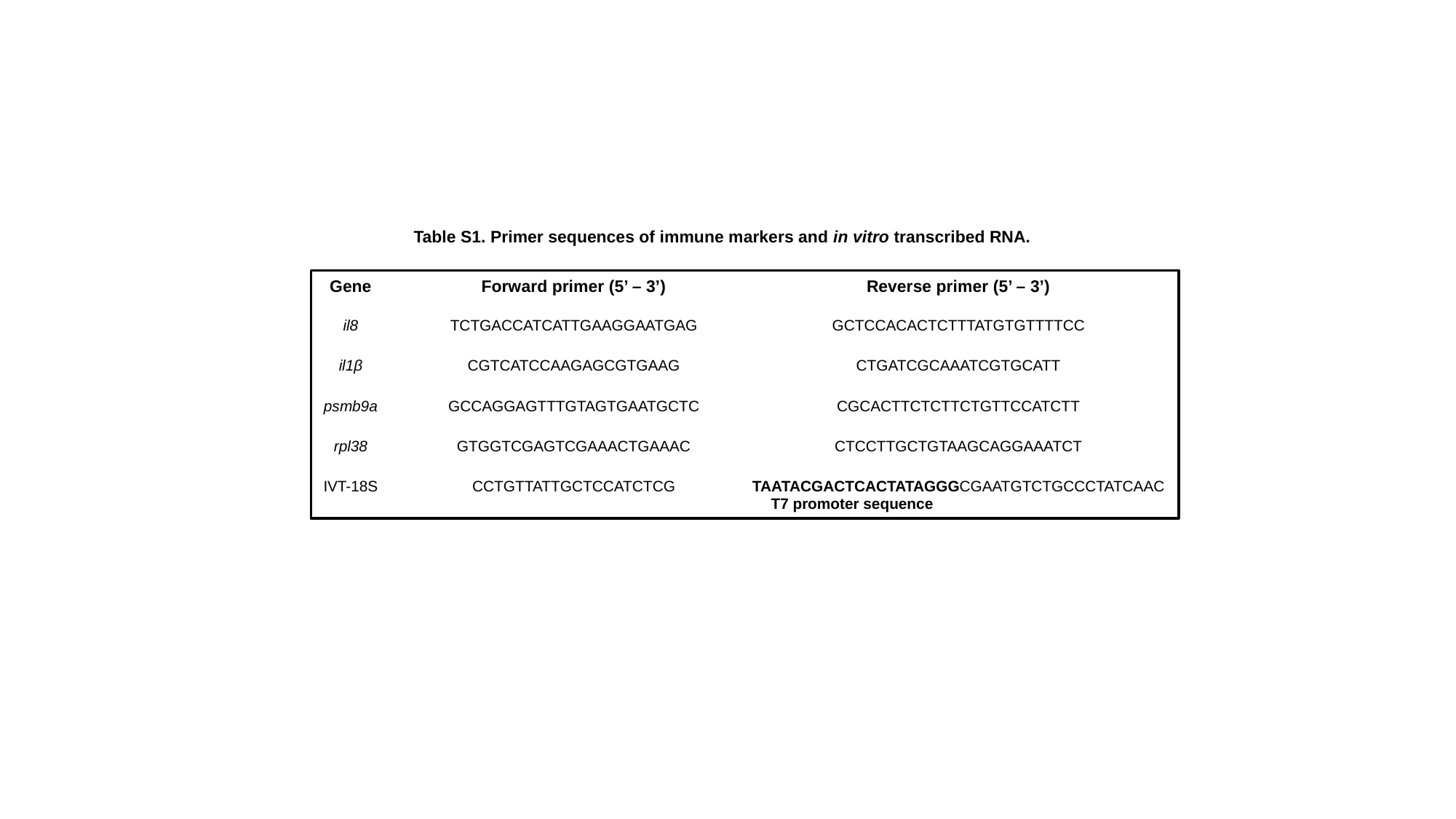

Table S1. Primer sequences of immune markers and in vitro transcribed RNA.
| Gene | Forward primer (5’ – 3’) | Reverse primer (5’ – 3’) |
| --- | --- | --- |
| il8 | TCTGACCATCATTGAAGGAATGAG | GCTCCACACTCTTTATGTGTTTTCC |
| il1β | CGTCATCCAAGAGCGTGAAG | CTGATCGCAAATCGTGCATT |
| psmb9a | GCCAGGAGTTTGTAGTGAATGCTC | CGCACTTCTCTTCTGTTCCATCTT |
| rpl38 | GTGGTCGAGTCGAAACTGAAAC | CTCCTTGCTGTAAGCAGGAAATCT |
| IVT-18S | CCTGTTATTGCTCCATCTCG | TAATACGACTCACTATAGGGCGAATGTCTGCCCTATCAAC |
T7 promoter sequence
